# Microbiome Continuum: Connecting Environmental, Host‐Associated and Engineered Microbiomes Through One Health and Artificial Intelligence

**DOI:** 10.1111/1758-2229.70445

**Published:** 2026-10-23

**Authors:** Mohammad Nazrul Islam Bhuiyan

**Affiliations:** ^1^ BCSIR Chattogram Laboratories BCSIR Chattogram Bangladesh; ^2^ Institute of Food Science and Technology Bangladesh Council of Scientific and Industrial Research (BCSIR) Dhaka Bangladesh

**Keywords:** artificial intelligence, environmental microbiology, microbiome continuum, multi‐omics, one health

## Abstract

Microbiomes are increasingly understood as interconnected ecological systems spanning soil, water, plants, animals, humans and engineered environments rather than as isolated host‐associated communities. This microbiome continuum provides a mechanistic perspective for One Health by linking microbial cells with the movement of metabolites, genes, signalling molecules and ecological functions across environmental boundaries. Here, we critically examined the biological processes that maintain this continuity, including dispersal, horizontal gene transfer (HGT), metabolic exchange, environmental exposure and host–microbe communication. We consider how climate change and anthropogenic disturbance reshape these interactions and influence antimicrobial resistance (AMR), non‐communicable disease, ecosystem function and pathogen emergence. We further evaluate how artificial intelligence (AI), including machine learning and deep learning, can integrate multi‐omics and longitudinal data, while emphasizing that predictive performance does not establish causality and that AI remains dependent on data quality, biological knowledge and experimental validation. Engineered microbiomes offer opportunities for targeted intervention, but ecological stability, biosafety, HGT and translational uncertainty remain important constraints. We propose a One Health Microbiome Intelligence Framework (OH‐MIF) as a conceptual architecture for cross‐domain integration, with emphasis on standardized data, causal validation, equitable participation and human oversight.

## Graphical abstract

The microbiome continuum links environmental, host‐associated and engineered microbiomes through the exchange of microorganisms, genes, metabolites and biological functions. Integration of multi‐omics and artificial intelligence (AI) enables cross‐domain analysis and predictive insights, supporting One Health approaches to healthier environments, animals and people, and to a more sustainable future.

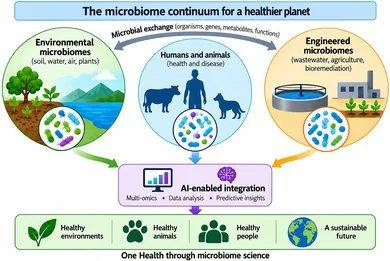

## 1 Introduction

Microorganisms are fundamental components of virtually every ecosystem, but microbiome research has historically been organized around relatively discrete compartments: clinical microbiomes, agricultural microbiomes, environmental microbiomes and engineered microbial systems. This organization has been useful for developing specialized methods and disciplines, yet it can obscure the ecological continuity through which microorganisms, genes, metabolites and environmental conditions connect these domains. The human microbiome, for example, is shaped not only by host physiology but also by diet, environmental exposures, antimicrobial use, built environments and interactions with microbial reservoirs outside the host (Ahn and Hayes 2021; Lynch and Pedersen 2016). The resulting biological system is therefore better understood as part of a larger continuum rather than as an autonomous microbial compartment.

The microbiome continuum captures this cross‐scale connectivity by treating environmental, host‐associated and engineered microbiomes as interacting components of a broader ecological network. This perspective aligns with the One Health framework, which recognizes that human, animal and environmental health are interdependent (Destoumieux‐Garzón et al. 2018; Trinh et al. 2018). Recent international initiatives have further emphasized the need to integrate microbiomes across human, plant, animal and planetary health rather than treating them as independent research subjects (Affagard et al. 2026; Proctor et al. 2025). The microbiome continuum therefore provides a biological substrate for One Health rather than simply another organizational label.

A central implication is that ecological continuity does not require direct microbial colonization. Microorganisms can influence another ecosystem through dispersal, but influence can also be transferred through microbial metabolites, extracellular products, signalling molecules, mobile genetic elements, altered nutrient availability and environmental physicochemical changes. Soil‐derived environmental exposures, for example, can interact with host‐associated microbial communities even when durable colonization by environmental organisms is uncertain (Ahn and Hayes 2021; Ooi et al. 2020; Roslund et al. 2024). Microbial metabolites provide another potentially important bridge because they represent functional outputs of microbial activity that can move through food webs, water, aerosols, host tissues or environmental compartments. The continuum should therefore be conceptualized as a network of organisms, functions and molecular flows, not simply a map of microbial migration.

Technological advances have made this conceptual shift increasingly tractable. Metagenomics provides information about genetic potential, whereas metatranscriptomics, metaproteomics and metabolomics provide complementary information about activity and biochemical function (Wishart 2019; Alves et al. 2025, 2026). Large‐scale microbiome initiatives have demonstrated the breadth of microbial diversity across Earth, while improved analytical standards have highlighted the importance of sampling design, metadata, laboratory procedures and computational reproducibility (Thompson et al. 2017; Knight et al. 2018). Nevertheless, the increasing dimensionality of microbiome datasets also exposes a major analytical challenge: associations among taxa, genes, metabolites and host phenotypes are numerous, context dependent and frequently non‐causal.

Artificial intelligence (AI), including machine learning (ML), deep learning (DL) and related computational approaches, provides tools for identifying patterns in such high‐dimensional datasets. Recent microbiome studies have used ML, graph‐based methods and deep neural networks for phenotype prediction and biomarker discovery (Aghdam et al. 2026; Shi, He, et al. 2026; Shi, Wang, et al. 2026). However, AI should be regarded as an analytical instrument rather than a substitute for biological reasoning. Predictive accuracy can arise from confounding variables, batch effects or population‐specific signatures and model performance in a discovery cohort does not establish generalizability or causality. The value of AI therefore depends on the quality and representativeness of the underlying data, the biological question being addressed and the availability of independent experimental validation.

This distinction is particularly important for a microbiome continuum because the most consequential biological relationships may not be captured by existing datasets. An algorithm trained primarily on taxonomic abundance may identify recurring associations but remain unable to detect an unmeasured metabolite‐mediated pathway. Similarly, models developed from well‐characterized human cohorts may perform poorly in tropical agricultural systems, low‐resource clinical settings or understudied environmental microbiomes. Over‐reliance on computational prediction could therefore reproduce existing methodological blind spots rather than discover genuinely new ecological mechanisms.

Against this background, this review examines the microbiome continuum as a mechanistic framework for One Health. We first consider environmental, host‐associated and engineered microbiomes and the processes connecting them. We then examine functional consequences for host physiology, non‐communicable disease, antimicrobial resistance (AMR) and climate‐sensitive microbial processes. Finally, we evaluate microbiome engineering and AI‐enabled analysis, emphasizing evidence boundaries, failure modes, ecological risks, equity and the requirements for translating a proposed One Health Microbiome Intelligence Framework into a reliable research and surveillance architecture.

## 2 Microbiome Continuum: Components and Mechanisms of Connectivity

### 2.1 Environmental Microbiomes as the Ecological Foundation

Environmental microbiomes constitute the largest and most heterogeneous component of the microbiome continuum. Soil, freshwater, marine systems, sediments, plants, aerosols and built environments contain microbial communities that regulate nutrient transformation, decomposition, carbon cycling, nitrogen cycling and ecosystem productivity (Cavicchioli et al. 2019; Tiedje et al. 2022). Their influence extends beyond ecosystem boundaries because environmental microorganisms and their products are continuously exchanged with plants, animals and humans.

Soil provides an especially important example of this continuity. Soil microbial communities participate in nutrient availability, plant–microbe interactions organic‐matter transformation and ecosystem resilience (Nadeu et al. 2023; Wang et al. 2024). Agricultural practices can substantially alter soil microbiome assembly and microbial interactions, demonstrating that microbial community structure is responsive to land management rather than being a fixed property of soil (Mo et al. 2024). Metagenomic and culturomic approaches are increasingly revealing the functional complexity of these communities and their potential relevance to sustainable agriculture (Clagnan et al. 2024; Jagadesh et al. 2024).

The significance of environmental microbiomes for human health is not limited to pathogen exposure. Environmental microbial diversity may influence host‐associated microbial communities through repeated exposure, dietary pathways and environmental intermediates (Ahn and Hayes 2021; Ooi et al. 2020). The soil–plant–food–gut pathway is particularly relevant because agricultural microbiomes influence the microbial and chemical composition of food before consumption. This provides a plausible route through which land management, environmental contamination and microbial ecology can become biologically connected to host‐associated microbiomes without requiring direct establishment of environmental taxa within the host.

Marine and freshwater microbiomes similarly contribute to the continuum through nutrient cycling, food webs and exposure pathways. Microbial responses to warming, acidification, oxygen depletion and pollution can alter ecosystem functions and potentially affect human and animal health through changes in food webs, water quality and pathogen ecology (Cavicchioli et al. 2019; Hutchins et al. 2019; Webster 2026). Thus, environmental microbiomes should be regarded not simply as reservoirs of microorganisms but as dynamic functional systems whose outputs can propagate across ecological boundaries.

### 2.2 Host‐Associated Microbiomes as Responsive Interfaces

Host‐associated microbiomes represent dynamic interfaces between environmental exposure and physiological function. The human intestinal microbiome contributes to nutrient transformation, immune development, epithelial integrity and host metabolism, but these functions are strongly influenced by diet, medication, environmental exposure and host physiology (Lynch and Pedersen 2016). Similar ecological principles apply to animal, plant and other host‐associated microbiomes.

A mechanistic interpretation requires moving beyond taxonomic abundance towards microbial function. Short‐chain fatty acids (SCFAs), including acetate, propionate and butyrate, illustrate this principle. These metabolites arise largely through microbial fermentation and can influence epithelial integrity, immune signalling and host metabolism (Du et al. 2024; Parada Venegas et al. 2019). Tryptophan‐derived microbial metabolites provide another route of communication by influencing host signalling pathways, including aryl hydrocarbon receptor‐dependent processes (Agus et al. 2018; Roager and Licht 2018; Waclawiková and El Aidy 2018). Such pathways demonstrate why metabolite flow can be more informative than microbial presence alone when investigating ecological continuity.

The microbiome also participates in multi‐organ communication. Gut‐derived microbial metabolites and microbial products influence the gut–brain, gut–liver and gut–skin axes, while host physiology reciprocally modifies microbial composition and function (Dutta et al. 2026; Shi and Chen 2026; Zhang, Zhang, et al. 2026). Recent work further highlights interactions between microbiota and immune responses in cancer and other chronic disorders (Elsheikh et al. 2026; Gorji et al. 2026; Su et al. 2026). However, associations between microbial signatures and disease should not automatically be interpreted as evidence that dysbiosis is a primary cause. Disease‐associated changes can reflect altered diet, medication, inflammation, tissue physiology or treatment exposure. Distinguishing cause, consequence and compensation remains one of the central challenges of microbiome science.

### 2.3 Engineered Microbiomes as Designed Components of the Continuum

Engineered microbiomes occupy a distinctive position because their composition or function is intentionally modified. Synthetic biology, metabolic engineering and genome editing can alter individual strains, construct synthetic communities or reshape existing microbial ecosystems (Aggarwal et al. 2023; Ciurea et al. 2026). Recent work on programmable cell–cell adhesion illustrates how engineering can alter spatial organization and productivity within synthetic microbial communities (Chen et al. 2026).

In health applications, engineered microorganisms are being investigated as living therapeutics, biosensors and vehicles for localized production of therapeutic molecules (Ding et al. 2026; Sabio et al. 2026). Engineered microbial communities may also provide complementary metabolic functions that are difficult to obtain from a single strain. Yet these systems remain biologically and operationally demanding. Community stability, genetic drift, host specificity, manufacturing consistency, containment and horizontal gene transfer (HGT) are all relevant risks.

Environmental engineering provides a complementary perspective. Wastewater treatment, anaerobic digestion, constructed wetlands and related systems depend on microbial communities whose collective metabolism determines process performance. Genomics and molecular surveillance can improve characterization of these systems, while wastewater‐based epidemiology can provide population‐level information about infectious disease and AMR (Cajas‐Corrales et al. 2025). In this context, engineered microbiomes are not isolated technological platforms; they are interfaces between environmental microbial ecology and public health.

The resulting continuum is summarized in Table 1, which distinguishes the principal microbiome compartments by dominant functions, mechanisms of cross‐domain influence and evidence requirements. The conceptual relationships are shown in Figure 1.

**TABLE 1:** Functional organization of the microbiome continuum and mechanisms of cross‐domain influence.

Microbiome domain	Representative functions	Principal routes of continuity	Key evidence requirement	References
Soil and terrestrial microbiomes	Nutrient cycling, decomposition, plant interactions, carbon transformation	Microbial dispersal, plant–soil exchange, metabolites, food pathways	Longitudinal field measurements linking community/function to ecosystem outcomes	Nadeu et al. (2023), Mo et al. (2024), Clagnan et al. (2024) and Jagadesh et al. (2024)
Aquatic and marine microbiomes	Carbon and nutrient cycling, food‐web regulation, contaminant transformation	Waterborne dispersal, metabolites, trophic transfer, environmental reservoirs	Environmental gradients plus functional and biogeochemical measurements	Cavicchioli et al. (2019), Hutchins et al. (2019) and Tiedje et al. (2022)
Plant microbiomes	Nutrient acquisition, stress responses, pathogen interactions	Soil–plant exchange, root exudates, agricultural management	Field validation across soils, crops and climate conditions	Jagadesh et al. (2024), Sharma et al. (2025) and Wang et al. (2024)
Animal and human microbiomes	Metabolism, immune regulation, barrier function, host signalling	Food, water, environmental exposure, metabolites, microbial products	Mechanistic and longitudinal host studies	Lynch and Pedersen (2016), Ahn and Hayes (2021) and Muhummed et al. (2025)
Engineered microbiomes	Bioproduction, bioremediation, biosensing, therapeutics	Designed strains, synthetic consortia, engineered genetic functions	Stability, containment, efficacy and ecological risk assessment	Aggarwal et al. (2023), Chen et al. (2026), Ding et al. (2026) and Sabio et al. (2026)
Wastewater and engineered treatment systems	Pollutant removal, nutrient recovery, surveillance	Waste streams, mobile genes, microbial products and molecular signals	Standardized sampling, longitudinal surveillance and process‐level validation	Cajas‐Corrales et al. (2025) and Ece et al. (2026)

**FIGURE 1:**
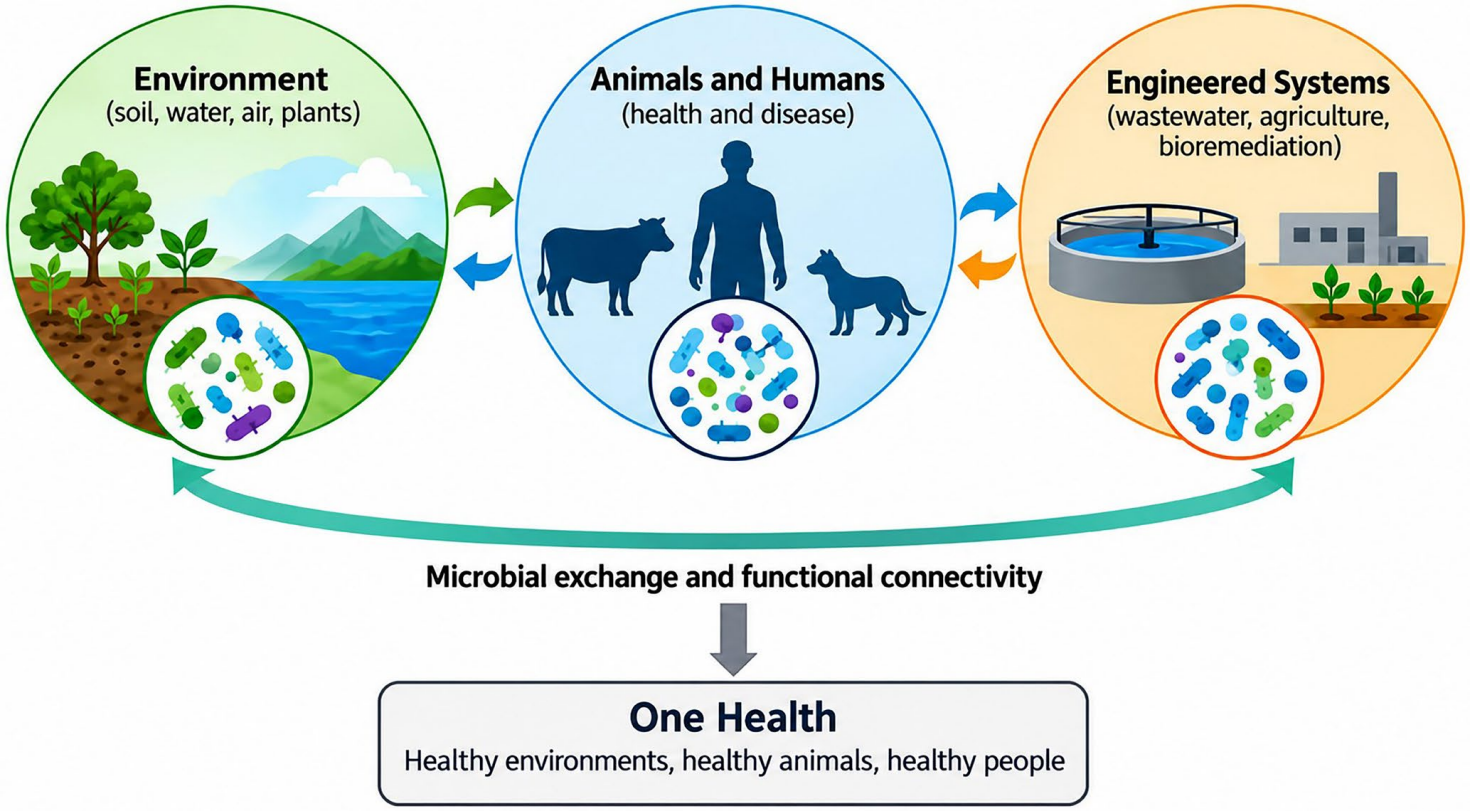
The microbiome continuum across One Health domains.

Conceptual representation of environmental, host‐associated and engineered microbiomes as interconnected ecological systems. Connectivity is represented through four distinct pathways: (i) movement of microorganisms by dispersal and host‐associated transmission; (ii) movement of microbial genes and mobile genetic elements; (iii) transfer of microbial metabolites, extracellular products and signalling molecules and (iv) modification of ecological conditions that alter community assembly and function. The figure emphasizes that ecological influence does not require stable colonization and that engineered microbiomes form part of the continuum rather than an independent technological category. One Health provides the integrative context linking human, animal and environmental consequences. The figure is conceptual and does not imply equal magnitude or direction of exchange among all compartments.

## 3 Functional Consequences of Microbiome Continuity

### 3.1 From Microbial Composition to Microbial Function

A major limitation of taxon‐centred microbiome analysis is that community composition alone does not reliably predict functional output. Distinct microbial communities may perform similar functions through functional redundancy, whereas similar communities can express different metabolic activities under different environmental conditions. Consequently, understanding the microbiome continuum requires moving beyond who is present towards what functions are active and how those functions propagate across domains.

Metaproteomics and metabolomics provide complementary approaches to this functional perspective. Metaproteomics captures proteins represented within microbial communities, whereas metabolomics characterizes biochemical outputs arising from microbial and host metabolism (Alves et al. 2025, 2026; Wishart 2019). Their integration can therefore identify functional intermediates linking environmental conditions, microbial activity and host responses. This is particularly relevant to microbiome continuity because cross‐domain influence may occur without persistent microbial colonization, through metabolites, exchanged substrates, signalling molecules or genetic material.

Several microbial processes illustrate this functional connectivity (Table 2). Carbon and nitrogen transformations connect microbial activity with ecosystem productivity, carbon storage, nutrient availability and greenhouse‐gas fluxes, although their magnitude remains strongly context dependent (Cavicchioli et al. 2019; Crowther et al. 2024; Jansson 2023; Tiedje et al. 2022; Toor et al. 2024). In host‐associated systems, microbial production of SCFAs such as acetate, propionate and butyrate can influence epithelial, immune and metabolic signalling, although the causal contribution of individual metabolites to specific diseases remains heterogeneous (Du et al. 2024; Parada Venegas et al. 2019; Zhang, Pi, et al. 2026). Similarly, tryptophan transformation generates indole and related metabolites that participate in immune, epithelial and neurochemical signalling, providing a biochemical route through which microbial metabolism can influence host physiology (Agus et al. 2018; Roager and Licht 2018; Waclawiková and El Aidy 2018).

**TABLE 2:** Microbial functions that provide mechanistic links across the microbiome continuum.

Functional process	Microbial output or activity	Potential cross‐domain consequence	Current evidence boundary	References
Carbon transformation	Decomposition, carbon fixation, microbial biomass formation	Soil carbon storage, greenhouse‐gas fluxes, ecosystem productivity	Strong ecological evidence; quantitative intervention effects remain context dependent	Cavicchioli et al. (2019), Crowther et al. (2024) and Jansson (2023)
Nitrogen transformation	Nitrification and denitrification	Nitrogen availability and nitrous oxide production	Strong mechanistic basis; magnitude depends on environmental conditions	Tiedje et al. (2022) and Toor et al. (2024)
SCFA production	Acetate, propionate, butyrate	Epithelial, immune and metabolic signalling	Mechanistic evidence is substantial, but disease‐specific causal effects vary	Du et al. (2024), Parada Venegas et al. (2019) and Zhang, Pi, et al. (2026)
Tryptophan transformation	Indoles and other microbial metabolites	Immune, epithelial and neurochemical signalling	Strong biochemical pathways; clinical causality remains heterogeneous	Agus et al. (2018), Roager and Licht (2018) and Waclawiková and El Aidy (2018)
HGT	Movement of mobile genetic elements	Dissemination of AMR and other traits	Mechanistically established; ecological contribution varies by environment	Chigozie et al. (2025) and Fakhri et al. (2026)
Metabolic cross‐feeding	Exchange of substrates and products among taxa	Community stability and emergent ecosystem functions	Well‐established ecological principle; difficult to quantify in complex natural systems	Banerjee et al. (2018) and Zhou et al. (2024)
Plant–microbe interactions	Nutrient exchange, signalling, stress modulation	Crop productivity and environmental resilience	Strong experimental and ecological evidence; field reproducibility remains context dependent	Jagadesh et al. (2024), Sharma et al. (2025) and Wang et al. (2024)

Other processes operate primarily through ecological and genetic connectivity. Metabolic cross‐feeding, in which microbial taxa exchange substrates and metabolic products, can support community stability and emergent functions but remains difficult to quantify in complex natural ecosystems (Banerjee et al. 2018; Zhou et al. 2024). HGT provides another potential continuum‐wide pathway by which mobile genetic elements can disseminate AMR and other traits, although its ecological contribution varies substantially among environments (Chigozie et al. 2025; Fakhri et al. 2026). In agricultural systems, plant–microbe interactions integrate nutrient exchange, signalling and stress modulation with crop productivity and environmental resilience, while field‐level reproducibility remains context‐dependent (Jagadesh et al. 2024; Sharma et al. 2025; Wang et al. 2024).

Together, these examples indicate that microbiome continuity is fundamentally a functional rather than purely taxonomic phenomenon. The strongest evidence currently exists for established biochemical and ecological processes, whereas the magnitude and causal consequences of cross‐domain effects are often context dependent. This distinction is important for One Health research: detecting a shared microbial taxon across environments may demonstrate connectivity, but demonstrating a shared function, metabolite or genetic trait and its causal relevance provides stronger evidence for biological continuity.

### 3.2 Microbiome, Non‐Communicable Disease and Causality

The relationship between microbiomes and non‐communicable diseases illustrates both the potential and the limitations of continuum thinking. Obesity, metabolic disorders, cardiovascular disease, cancer and neurological disorders have all been associated with changes in microbial composition or function (Ahn and Hayes 2021; Huang et al. 2023; O'Toole and Paoli 2023). Yet the same phenotype can be associated with different microbial configurations across populations, and microbial changes can arise from diet, medication, inflammation and disease itself.

Microbial metabolites provide more mechanistically informative endpoints than taxonomic signatures alone. SCFAs can influence epithelial and immune function, while tryptophan metabolites and bile‐acid derivatives participate in host signalling (Agus et al. 2018; Du et al. 2024; Roager and Licht 2018). These observations support a model in which environmental and dietary inputs are transformed by microbial communities into chemical signals that influence host physiology. Nevertheless, demonstrating that a metabolite is biologically active does not by itself establish that altering the responsible microbial pathway will improve a clinical outcome.

The same caution applies to cancer. Microbial communities can influence inflammation, metabolism and immune responses and gut microbiome composition has been associated with responses to immune checkpoint inhibitors (Elsheikh et al. 2026; Gorji et al. 2026; Su et al. 2026). However, the reproducibility of response‐associated microbial signatures across tumour types, treatment regimens and populations remains a translational challenge. Consequently, microbiome‐based biomarkers should be evaluated not only by discrimination within a dataset but also by biological plausibility, external validation, calibration and prospective clinical utility.

### 3.3 AMR as a Continuum Phenomenon

AMR illustrates particularly clearly why microbiome compartments cannot be considered independently. Resistant microorganisms and resistance determinants occur in human‐associated, animal, agricultural, soil, aquatic and wastewater microbiomes. Antimicrobial exposure creates selection pressure, while HGT can facilitate movement of resistance determinants among microbial populations (Chigozie et al. 2025; Fakhri et al. 2026).

The ecological importance of AMR is therefore broader than clinical resistance alone. Wastewater, agricultural runoff and environmental contamination can provide interfaces where resistant organisms, mobile genetic elements and selective pressures converge. One Health surveillance consequently requires coordinated measurement across human, animal and environmental reservoirs (Affagard et al. 2026; Proctor et al. 2025). Wastewater‐based epidemiology and genomic surveillance provide potentially useful components of such systems, but global standardization remains necessary for reliable cross‐site comparison (Cajas‐Corrales et al. 2025).

The continuum also provides a rationale for exploring microbiome‐derived antimicrobial strategies. Bacteriocins offer one example of naturally occurring microbial antagonism, while bacteriophages and lysins provide additional approaches for selectively targeting bacterial pathogens (Arbulu and Kjos 2024; Briers 2026; Soltani et al. 2021). However, narrow host range, resistance evolution, manufacturing and regulatory requirements mean that these approaches should not be presented as universal replacements for antibiotics. Their value will depend on defined indications, validated efficacy and ecological consequences.

## 4 Climate Change as a Microbiome‐Continuum Perturbation

Climate change affects microbial ecosystems through temperature, precipitation, oxygen availability, ocean acidification, extreme weather and changes in resource availability. Because microorganisms regulate decomposition, nutrient cycling and greenhouse‐gas fluxes, climate‐driven microbial changes can generate feedbacks that extend beyond individual ecosystems (Cavicchioli et al. 2019; Guigard et al. 2025; Tiedje et al. 2022).

Soil microbiomes are particularly relevant because temperature and moisture influence microbial decomposition and carbon transformation. Agricultural practices further modify these responses by altering nutrient availability, soil structure and microbial community assembly (Mo et al. 2024; Sharma et al. 2025). The interaction between climate stress and agricultural management is especially important in tropical and subtropical regions, where heat, rainfall variability, flooding and soil degradation may occur simultaneously. For countries in the Global South, microbiome research should therefore be integrated with locally relevant agricultural, environmental and public‐health priorities rather than extrapolated exclusively from temperate datasets.

Marine microbiomes provide another climate‐sensitive component. Warming, acidification and deoxygenation alter microbial physiology and community structure, with implications for carbon cycling and marine productivity (Hutchins et al. 2019; Webster 2026). Such changes illustrate that the microbiome continuum is also a climate‐system continuum: microbial responses influence biogeochemical processes, which in turn affect the environmental conditions experienced by other microbial communities.

Microbial climate feedbacks include methane and nitrous oxide cycling. Methanogenic and methanotrophic communities regulate methane production and consumption, whereas nitrifying and denitrifying microorganisms contribute to nitrogen transformations and nitrous oxide fluxes (Cavicchioli et al. 2019; Tiedje et al. 2022). Agricultural management can modify these processes, making microbial ecology relevant to climate adaptation and mitigation. However, claims about microbiome‐based climate mitigation should remain evidence calibrated. Demonstrating that microorganisms participate in carbon or nitrogen cycling is not equivalent to demonstrating that deliberate microbiome manipulation produces a net climate benefit at field or regional scale.

Climate change also alters host‐associated microbiomes indirectly through food availability, environmental exposure, pollution and pathogen distribution (Guigard et al. 2025; Toor et al. 2024). Extreme weather can disrupt water and sanitation systems, modify microbial exposure pathways and increase opportunities for contamination. The microbiome continuum therefore provides a useful framework for studying climate‐related health risks without reducing them to a single microbial mechanism. These interconnected processes are summarized in Figure 2.

**FIGURE 2:**
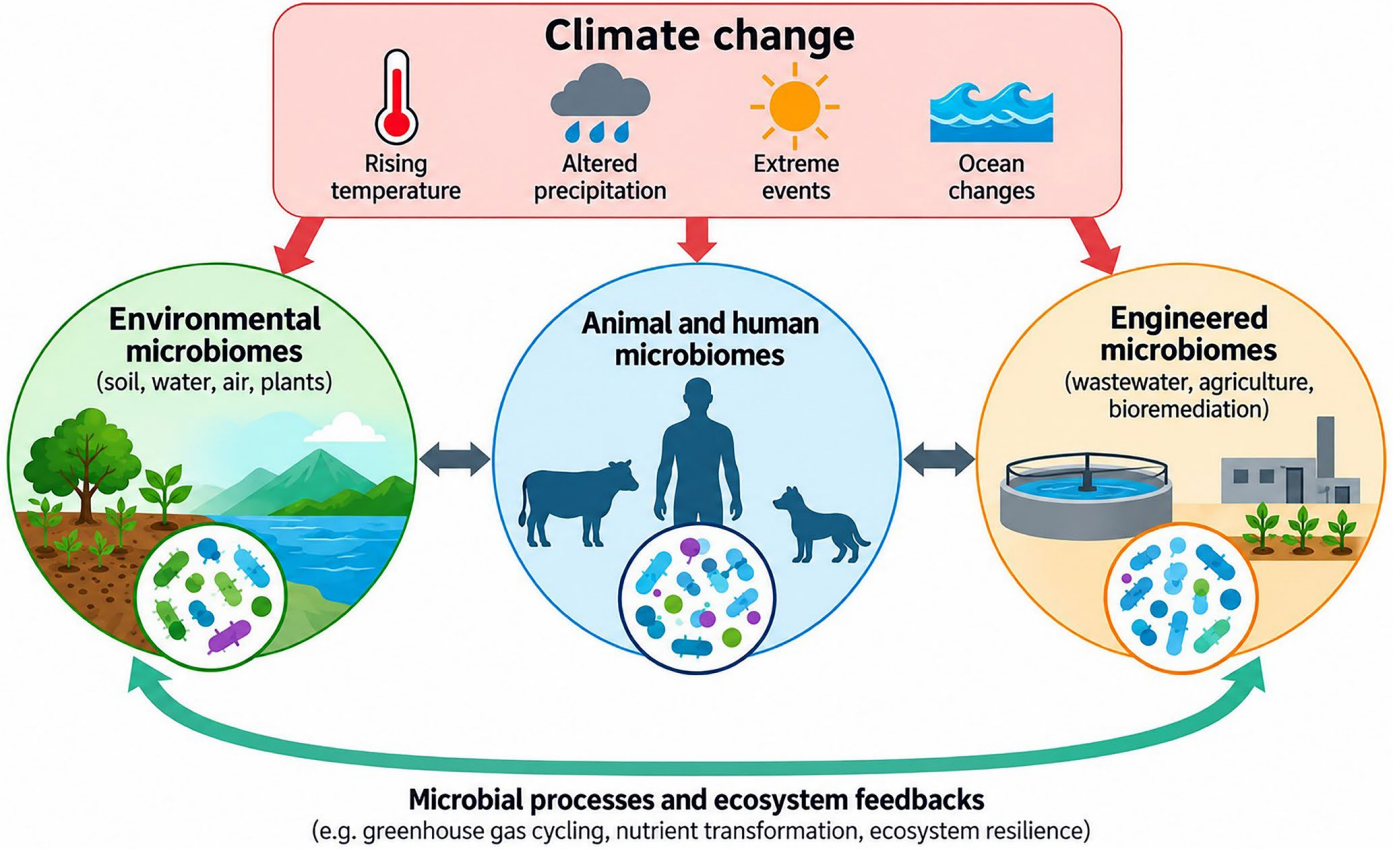
Climate‐driven perturbation and feedback across the microbiome continuum.

Conceptual representation of climate‐related drivers, including warming, altered precipitation, flooding and drought, ocean acidification, deoxygenation and changes in agricultural production acting on environmental, host‐associated and engineered microbiomes. The figure distinguishes direct microbial responses from downstream consequences involving carbon and nitrogen cycling, greenhouse‐gas fluxes, food and water systems, host–microbiome interactions and pathogen ecology. Dashed arrows represent potential feedbacks rather than quantified causal effects. The framework emphasizes that the magnitude and direction of microbial responses depend on ecosystem context, baseline community structure and interacting anthropogenic pressures.

## 5 Engineered Microbiomes: Opportunity Constrained by Ecology

Microbiome engineering is shifting microbial research from observation towards controlled modification. CRISPR‐based editing, metabolic engineering, genetic circuits and synthetic‐community design can alter microbial functions or ecological interactions (Aggarwal et al. 2023; Ciurea et al. 2026). Recent synthetic‐community studies demonstrate that engineering spatial organization and cell–cell interactions can influence community productivity (Chen et al. 2026).

In host‐associated applications, engineered bacteria are being investigated as living therapeutics capable of sensing environmental signals, producing therapeutic molecules or modifying local microbial metabolism (Ding et al. 2026; Sabio et al. 2026). These systems are conceptually attractive because they can combine sensing and response within living cells. Yet biological performance is not equivalent to engineering success. An engineered strain must remain functional in the target ecological environment, compete or cooperate appropriately with resident communities, avoid undesirable gene transfer and exhibit predictable behaviour over time.

Synthetic consortia introduce additional levels of complexity. Community members may depend on metabolic cross‐feeding, spatial organization or signalling interactions that are difficult to reproduce outside controlled laboratory conditions. The resulting system may also evolve after deployment. Therefore, translational development should evaluate community stability, functional persistence, genetic stability, host specificity and ecological containment rather than relying solely on short‐term efficacy.

Environmental engineering presents related challenges. Microbial consortia are already integral to wastewater treatment, bioremediation and agricultural systems. The distinction between an engineered microbiome and a managed natural microbiome can therefore be gradual rather than categorical. This supports the continuum concept but also means that ecological risk assessment must account for the receiving environment, not merely the engineered organism. Table 3 summarizes the principal opportunities and failure modes of emerging microbiome technologies.

**TABLE 3:** Emerging microbiome technologies: Evidence boundaries, opportunities and failure modes.

Technology	Demonstrated or established capability in literature	Principal limitation or failure mode	Required validation	References
Metagenomics	Community composition and genetic potential	Functional activity cannot be inferred directly from genes alone	Integration with activity and environmental measurements	Thompson et al. (2017) and Ece et al. (2026)
Metaproteomics	Protein‐level evidence of community function	Complex taxonomic assignment and quantitative uncertainty	Improved computational assignment and orthogonal validation	Alves et al. (2025, 2026)
Metabolomics	Functional biochemical outputs and microbial–host signalling	Attribution of metabolites to specific organisms/pathways	Targeted validation and mechanistic experiments	Wishart (2019) and Roager and Licht (2018)
ML/DL	Pattern recognition, classification and phenotype prediction	Overfitting, population specificity, interpretability and batch effects	Independent validation, calibration and biological testing	Aghdam et al. (2026) and Shi, He, et al. (2026) and Shi, Wang, et al. (2026)
Engineered probiotics	Programmable sensing or therapeutic functions	Stability, containment, host specificity and ecological interactions	Longitudinal efficacy and biosafety assessment	Ding et al. (2026) and Sabio et al. (2026)
Synthetic consortia	Coordinated metabolic functions	Community instability and manufacturing reproducibility	Ecological stability and functional reproducibility	Chen et al. (2026) and Aggarwal et al. (2023)
Bacteriophages/lysins	Targeted antibacterial activity	Resistance, host range and regulatory/manufacturing constraints	Strain‐specific efficacy and ecological assessment	Briers (2026) and Soltani et al. (2021)
Microbial environmental engineering	Bioremediation, treatment and resource recovery	Field variability and unintended ecological effects	Field‐scale and ecosystem‐level validation	Bux et al. (2026) and Cajas‐Corrales et al. (2025)

## 6 AI and Microbiome Research

### 6.1 AI as an Analytical Tool Rather Than a Replacement for Biological Reasoning

Microbiome research generates heterogeneous measurements spanning taxonomy, genes, transcripts, proteins, metabolites, host phenotypes and environmental variables. AI approaches can help identify multivariable patterns that are difficult to detect using conventional statistical approaches. However, the appropriate role of AI is to augment, not replace, experimental microbiology, ecological reasoning and critical appraisal.

ML approaches such as random forests, support‐vector machines and related algorithms can classify phenotypes and identify candidate microbial features. Recent studies have also explored deep neural networks and graph‐based architectures for longitudinal phenotype prediction and multi‐level microbiome representation (Shi, He, et al. 2026; Shi, Wang, et al. 2026). These approaches demonstrate computational capacity, but predictive performance must be interpreted in relation to dataset size, feature dimensionality, validation strategy, class imbalance and external generalizability.

DL can model non‐linear relationships and hierarchical feature structures, but greater model complexity does not automatically imply greater biological insight. A model may accurately classify samples while relying on features that are proxies for geography, sequencing batch, medication or other confounders. Explainability methods can help identify influential features, but attribution is not equivalent to mechanistic causation. Biological interpretation therefore requires experimental follow‐up.

Longitudinal modelling is particularly relevant to the microbiome continuum because microbial communities change over time. Temporal approaches can potentially identify trajectories rather than static signatures, but irregular sampling, missing data and temporal confounding remain important limitations (Shi, He, et al. 2026). Such models should therefore be evaluated using independent temporal cohorts whenever possible.

### 6.2 Multi‐Omics Integration: From Association Towards Mechanism

Multi‐omics integration offers an important route towards functional microbiome science. Metagenomics can identify metabolic potential, metaproteomics can identify expressed proteins and metabolomics can identify biochemical outputs. The integration of these layers can generate hypotheses about microbial pathways and host responses (Alves et al. 2025, 2026; Wishart 2019).

AI can assist this integration, but it is not indispensable. Multidimensional microbiome datasets have long been integrated using statistical modelling, network analysis and mechanistic systems biology. AI adds potentially useful capacity for nonlinear prediction and multimodal pattern recognition, but its contribution should be demonstrated relative to appropriate conventional baselines.

The distinction matters particularly for a One Health Microbiome Intelligence Framework (OH‐MIF). A genuine cross‐domain intelligence platform would require more than connecting datasets to an AI model. It would require harmonized metadata, compatible sampling frameworks, comparable laboratory procedures, explicit representation of environmental and host variables, uncertainty quantification, transparent provenance and independent validation across geographical and ecological contexts. Without these foundations, an integrated platform could simply aggregate heterogeneous data without producing biologically reliable knowledge.

### 6.3 AI‐Enabled Diagnostics and Therapeutics

Microbiome‐based diagnostic models are being developed for infectious and non‐communicable diseases, while AI‐assisted molecular diagnostics may improve the interpretation of complex microbial data (Autenrieth et al. 2026; Gupta et al. 2026). However, diagnostic value requires more than internal classification accuracy. Prospective performance, calibration, population diversity, analytical reproducibility and clinical utility must be established before implementation.

AI is also being explored for microbiome‐directed intervention design, including selection of candidate microbial targets and prediction of treatment responses (Fang et al. 2025; Sharma et al. 2026). Such approaches could eventually support individualized interventions, but the translational chain remains incomplete in many applications. A predicted target must first be shown to have a causal or therapeutically actionable role, and an intervention must demonstrate reproducible benefit under clinically relevant conditions. This evidence hierarchy is summarized in Table 4.

**TABLE 4:** AI applications in microbiome research: evidence boundary, failure modes and translation requirements.

Application	What current literature supports	Key failure mode	Minimum evidence needed for translation	References
Disease classification	Identification of microbial patterns associated with phenotypes	Confounding, overfitting and population specificity	Independent external validation and clinical utility assessment	Autenrieth et al. (2026) and Shi, Wang, et al. (2026)
Longitudinal prediction	Modelling of microbial trajectories and phenotype prediction	Irregular sampling, temporal confounding and limited cohort diversity	Independent longitudinal validation	Shi, He, et al. (2026)
Biomarker discovery	Prioritization of candidate taxa/pathways	Association mistaken for causality	Mechanistic experiments and prospective validation	Aghdam et al. (2026) and Liu, Zhu, et al. (2026)
Multi‐omics integration	Detection of relationships across molecular layers	Batch effects, missing modalities and incompatible scales	Standardized datasets, orthogonal validation and reproducibility testing	Alves et al. (2025, 2026) and Wishart (2019)
Precision therapeutics	Candidate target and response prediction	Biological non‐specificity and uncertain causal pathways	Controlled intervention studies and prospective validation	Fang et al. (2025) and Kumar et al. (2026)
AMR surveillance	Integration of genomic and environmental information	Incomplete environmental sampling and inconsistent standards	Cross‐domain surveillance and harmonized genomic methods	Cajas‐Corrales et al. (2025) and Chigozie et al. (2025)
Digital‐twin concepts	Computational simulation of complex systems	Incomplete biological knowledge, data sparsity and validation burden	Prospective model testing against observed interventions	Ayivi‐Tosuh et al. (2026)
OH‐MIF	Conceptual integration of human, animal, agricultural and environmental data	Lack of interoperable cross‐domain datasets	Standardized data architecture, provenance, uncertainty and independent cross‐domain validation	Affagard et al. (2026) and Proctor et al. (2025)

## 7 The Limitations of AI‐Driven Microbiome Intelligence

### 7.1 Causality Remains the Central Biological Challenge

The microbiome field contains many statistically significant associations, but associations are not necessarily causal. A disease can alter the intestinal environment, which subsequently alters microbial composition; a medication can simultaneously influence host physiology and microbial communities and dietary changes can influence both disease risk and microbiome structure. Consequently, predictive models can perform well while remaining mechanistically ambiguous.

Causal inference approaches, including network models and Mendelian randomization, can help distinguish candidate causal relationships from simple associations, but they do not eliminate the need for experimental validation (Aghdam et al. 2026; Liu, Huang, et al. 2026). Stronger evidence requires triangulation among longitudinal observations, perturbation experiments, mechanistic assays and independent cohorts.

### 7.2 Reproducibility and Batch Effects

Microbiome datasets are particularly sensitive to technical variation. Sample collection, storage, extraction procedures, sequencing platforms, laboratory batches, bioinformatic pipelines and metadata completeness can all influence observed microbial profiles (Knight et al. 2018; Ece et al. 2026). These effects can become embedded within AI models and produce apparently strong but non‐generalizable predictions.

Therefore, external validation should not be treated as an optional final step. It should be incorporated into model development from the outset. Where feasible, models should be challenged with samples collected at different times, in different laboratories and across different populations. Traditional statistical models and alternative AI architectures can also be compared to determine whether conclusions are method‐dependent.

### 7.3 Avoiding Over‐Reliance on AI

The rapid expansion of AI creates a methodological risk of its own. If researchers increasingly rely on automated systems for hypothesis generation, study design, coding, data processing, interpretation and scientific writing, human oversight may become weaker precisely where biological ambiguity is greatest. This risk is particularly important for understudied environments, where training data are sparse and established assumptions may not hold.

A robust microbiome‐AI workflow should therefore preserve independent human evaluation at critical decision points. Researchers should inspect data quality, compare alternative analytical approaches, examine biologically plausible and implausible predictions and experimentally test important model‐derived hypotheses. AI should increase analytical capacity without becoming an unquestioned authority.

### 7.4 The Danger of Methodological Circularity

An additional concern is that AI can reinforce the methods used to generate its training data. If current microbiome research primarily measures taxa, an AI model may become highly effective at predicting outcomes from taxonomic signatures while overlooking metabolites, spatial organization, ecological interactions or unmeasured environmental variables. The result may be improved prediction without corresponding expansion of biological understanding.

This is why the microbiome continuum requires methodological innovation alongside computational innovation. Metabolomics, metaproteomics, spatial microbiology, culturomics, ecological experiments and longitudinal environmental sampling are necessary complements to AI. The objective should be to discover new biological variables, not merely to model existing ones more efficiently.

## 8 One Health Microbiome Intelligence Framework (OH‐MIF)

The proposed OH‐MIF should be regarded as a conceptual architecture rather than an established operational platform. Its central purpose is to connect microbiome observations across human, animal, plant, agricultural and environmental systems while preserving information about context, uncertainty and biological provenance.

A functional OH‐MIF would contain at least five layers: observation, harmonization, inference, validation and translation. Observation would capture standardized molecular, ecological, clinical and environmental measurements. Harmonization would align metadata, sampling frameworks and analytical pipelines. Inference would use statistical, mechanistic and AI approaches to identify relationships. Validation would test predictions through independent datasets and experimental perturbation. Translation would determine whether validated findings can support clinical, agricultural or environmental decisions.

This structure addresses an important limitation of treating AI as the central component of One Health intelligence. AI should operate within a broader evidence architecture rather than define it. The most reliable system would combine conventional statistics, mechanistic modelling, ecological theory and AI, selecting the analytical method according to the biological question.

The OH‐MIF concept also requires explicit representation of uncertainty. A prediction generated from a dataset dominated by one geographical region should not be interpreted as universally applicable. Similarly, an association identified from human faecal samples cannot automatically be generalized to soil, wastewater or animal microbiomes. Cross‐domain integration therefore requires metadata that preserve ecological context rather than flattening different environments into a single data space. Figure 3 summarizes this revised pipeline.

**FIGURE 3:**
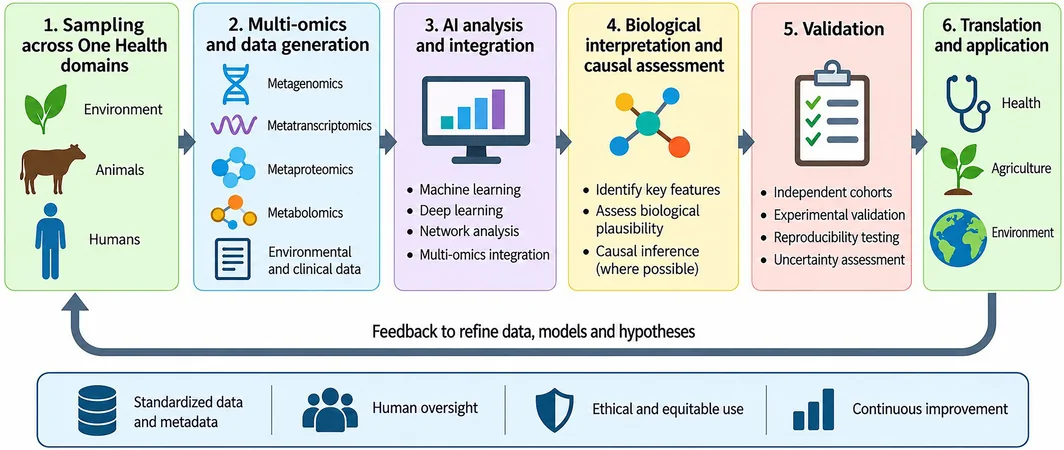
A validation‐centred AI pipeline for One Health microbiome research.

The framework begins with standardized sampling across environmental, animal, plant and human systems, followed by quality‐controlled multi‐omics and environmental measurements. AI, including ML, DL, network analysis and temporal modelling, operates as one analytical layer alongside conventional statistics and mechanistic modelling. Candidate relationships are then subjected to biological interpretation, causal assessment, independent validation and experimental testing. Only validated findings proceed to clinical, agricultural or environmental translation. OH‐MIF is represented as the cross‐domain data architecture connecting these stages. The framework explicitly includes human oversight, uncertainty assessment, alternative‐model comparison and feedback from failed predictions, thereby preventing computational outputs from being treated as inherently causal or universally transferable.

## 9 Global South Dimensions and Equity

The microbiome continuum is inherently global, yet microbiome datasets remain unevenly distributed across populations and environments. This matters because microbial communities are shaped by diet, climate, sanitation, antimicrobial exposure, urbanization, agriculture and socioeconomic conditions. A model trained predominantly on populations from high‐income countries may therefore fail to represent microbial ecology in other regions.

This limitation is especially relevant to South Asia and other tropical regions. Agricultural microbiomes in these settings experience combinations of heat, seasonal flooding, drought, intensive cultivation and variable soil management. Such conditions may generate microbial dynamics that are poorly represented in datasets collected under temperate conditions. The same issue applies to AMR surveillance, where environmental contamination, antimicrobial access, sanitation infrastructure and healthcare systems differ substantially among regions.

African microbiome research has already highlighted the importance of microbiomics for precision medicine while also identifying infrastructure and representation challenges (Nkera‐Gutabara et al. 2022). Broader concerns regarding inclusivity and equity in human microbiome research reinforce the need for research partnerships that include populations contributing samples and ecological knowledge (Rogers et al. 2019). Educational initiatives and microbiome literacy may also contribute to long‐term capacity development (Kaur et al. 2026).

For OH‐MIF, equity therefore cannot be treated as an ethical add‐on after model development. Geographic representation, local laboratory capacity, data governance and equitable access to analytical infrastructure should be incorporated into the architecture from the beginning. International collaboration should prioritize reciprocal capacity building rather than extraction of samples or data from under‐resourced regions.

## 10 Regulatory, Ecological and Societal Considerations

Microbiome technologies occupy different regulatory categories depending on whether they involve foods, therapeutics, engineered organisms, environmental applications or diagnostic systems. A common framework is therefore unlikely to be sufficient for all microbiome interventions. Product identity, intended use, exposure route, biological activity and environmental release must be considered individually.

For engineered microbiomes, ecological risk requires particular attention. HGT, persistence beyond the intended site, community instability and unintended metabolic effects are plausible concerns (Aggarwal et al. 2023; Ciurea et al. 2026). These risks do not imply that engineered microbiomes should not be developed; rather, they define the evidence required before deployment.

The same principle applies to environmental microbiome engineering. A microbial intervention that improves contaminant removal under laboratory conditions may behave differently in a complex ecosystem because of competition, predation, dispersal and changing environmental conditions. Field‐scale validation and ecological monitoring are therefore essential.

AI introduces a parallel set of governance challenges involving data ownership, privacy, algorithmic bias, transparency and accountability. Microbiome data can potentially reveal information about health, lifestyle and environmental exposure. Governance frameworks should consequently address not only technical data security but also consent, data stewardship and equitable benefit sharing (Li et al. 2026; Nkera‐Gutabara et al. 2022).

## 11 Towards a Research Agenda for the Microbiome Continuum

The next phase of microbiome research should move from cataloguing communities towards experimentally testing cross‐domain mechanisms. Four priorities are particularly important.

First, studies should distinguish microbial transfer from functional transfer. Demonstrating that an organism is detectable in two ecosystems does not establish ecological influence, while a metabolite or genetic element may exert influence without persistent organismal colonization. Experimental designs should therefore measure organisms, genes, metabolites and environmental variables simultaneously where feasible.

Second, microbiome studies should prioritize longitudinal and perturbational designs. Cross‐sectional associations are valuable for hypothesis generation but are insufficient for establishing temporal direction or causal mechanisms. Controlled interventions, natural experiments and repeated sampling can strengthen inference.

Third, AI studies should report model development and validation transparently. Relevant information includes cohort composition, feature generation, missing‐data handling, training strategy, hyperparameter selection, validation design, performance uncertainty, calibration and external testing. Models should be compared with appropriate non‐AI baselines, particularly where the added value of computational complexity remains uncertain.

Fourth, microbiome engineering should adopt ecological rather than purely molecular success criteria. A successful engineered microbiome should be evaluated for functional stability, containment, evolutionary robustness, ecological compatibility and reproducibility, not only for whether a desired gene or pathway is expressed.

These priorities converge on a broader principle: the microbiome continuum should become a testable ecological framework rather than a metaphor for connectivity. Its scientific value will ultimately depend on whether researchers can identify measurable mechanisms linking environmental perturbation, microbial function and host or ecosystem outcomes.

## 12 Conclusion

The microbiome continuum provides a useful framework for understanding microbial life as an interconnected ecological system spanning environmental, host‐associated and engineered ecosystems. Its central contribution is to expand the concept of microbial connectivity beyond organismal colonization to include metabolites, genes, signalling molecules and ecological functions. This perspective strengthens the biological foundation of One Health while exposing important uncertainties in causal inference, reproducibility, ecological translation and global representation. AI can enhance microbiome analysis by integrating complex datasets and generating testable predictions, but it should remain embedded within experimental microbiology, ecological theory and human oversight. Engineered microbiomes similarly offer new opportunities while requiring rigorous assessment of stability, biosafety and environmental consequences. The proposed OH‐MIF provides a conceptual route towards cross‐domain microbiome intelligence, but its implementation will depend on standardized data, representative sampling, causal validation, transparent governance and equitable research capacity. The future of microbiome science therefore lies not simply in larger datasets or more powerful algorithms, but in experimentally testing the mechanisms that connect microbial ecosystems across people, animals and the environment.

## Author Contributions

**Mohammad Nazrul Islam Bhuiyan:** conceptualization, visualization, resources, writing – review and editing, writing – original draft.

## Funding

The author has nothing to report.

## Ethics Statement

The author has nothing to report.

## Consent

The author has nothing to report.

## Conflicts of Interest

The author declares no conflicts of interest.

## Data Availability

Data sharing not applicable to this article as no datasets were generated or analysed during the current study.
